# Priorities among effective clinical preventive services in British Columbia, Canada

**DOI:** 10.1186/s12913-022-07871-0

**Published:** 2022-04-26

**Authors:** Hans Krueger, Sylvia Robinson, Trevor Hancock, Richard Birtwhistle, Jane A. Buxton, Bonnie Henry, Jennifer Scarr, John J. Spinelli

**Affiliations:** 1 H. Krueger & Associates Inc., Delta, Canada; 2 School of Population and Public Health, University of British Columbia, Vancouver, Canada; 3 BC Ministry of Health, Victoria, Canada; 4 School of Public Health and Social Policy, University of Victoria, Victoria, Canada; 5 Department of Family Medicine and Public Health Sciences, Queen’s University, Kingston, Canada; 6 Canadian Task Force on Preventive Health Care, Ottawa, Canada; 7 BC Center for Disease Control, Vancouver, Canada; 8 Child Health BC, Provincial Health Services Authority, Vancouver, Canada

## Abstract

**Background:**

Despite the long-standing experience of rating the evidence for clinical preventive services, the delivery of effective clinical preventive services in Canada and elsewhere is less than optimal. We outline an approach used in British Columbia to assist in determining which effective clinical preventive services are worth doing.

**Methods:**

We calculated the clinically preventable burden and cost-effectiveness for 28 clinical preventive services that received a ‘strong or conditional (weak) recommendation for’ by the Canadian Task Force on Preventive Health Care or an ‘A’ or ‘B’ rating by the United States Preventive Services Task Force. Clinically preventable burden is the total quality adjusted life years that could be gained if the clinical preventive services were delivered at recommended intervals to a British Columbia birth cohort of 40,000 individuals over the years of life that the service is recommended. Cost-effectiveness is the net cost per quality adjusted life year gained.

**Results:**

Clinical preventive services with the highest population impact and best value for money include services that address tobacco use in adolescents and adults, exclusive breastfeeding, and screening for hypertension and other cardiovascular disease risk factors followed by appropriate pharmaceutical treatment. In addition, alcohol misuse screening and brief counseling, one-time screening for hepatitis C virus infection in British Columbia adults born between 1945 and 1965, and screening for type 2 diabetes approach these high-value clinical preventive services.

**Conclusions:**

These results enable policy makers to say with some confidence what preventive manoeuvres are worth doing but further work is required to determine the best way to deliver these services to all those eligible and to establish what supportive services are required. After all, if a clinical preventive service is worth doing, it is worth doing well.

## Background

The Canadian Task Force on the Periodic Health Exam (later re-named the Canadian Task Force on Preventive Health Care - CTFPHC) began to review and rate clinical preventive services (CPS) in 1976 [1], and the US Preventive Services Task Force (USPSTF) took up and further developed this work starting in 1984 [2]. Despite the long-standing experience of rating the evidence for CPS, the delivery of effective CPS in Canada and elsewhere is less than optimal [3–5]. Suggested reasons for this include health care providers’ lack of time, as well as the patient’s inability to find a provider and the lack of coordination across providers and settings [6, 7]. Yarnall estimated that 7.4 h of every primary care physician’s working day would be required to fully satisfy all the USPSTF ‘A’ and ‘B’ recommendations, based on a patient panel of 2500 with an age and sex distribution similar to that of the US population [8]. An absence of policy and supportive management and payment systems is another factor in health systems focused on acute care [9].

The optimal delivery of CPS has important benefits for the health of the population. One study estimated that between 75,000 and 140,000 deaths could be avoided annually in the United States by increasing the use of nine CPS [10] while another estimated a saving of 2.6 million quality-adjusted life years in a US birth cohort of 4 million if utilization rates increased from current levels to 90% for 20 CPS [11].

The HealthPartners Institute in the US has attempted to reconcile the value of CPS with a provider’s lack of time, by prioritising effective CPS [11–14]. They note that the greatest population health improvement in the US could be gained by prioritizing CPS that address tobacco use, obesity-related behaviours and alcohol misuse [11].

Faced with this information and the lack of provincial policy on clinical preventive services in BC, the Ministry of Health established the Clinical Prevention Policy Review (CPPR) in January of 2007. The review process involved establishing a broad-based CPPR Expert Advisory Committee (the Committee), including experts from the US, the CTFPHC, the BC Medical Association (now Doctors of BC), the Canadian College of Family Physicians and others; Dr. Hans Krueger was hired as the lead consultant for the Committee.

The review asked three seemingly simple questions: What preventive manoeuvres are worth doing, what is the best way to deliver what is worth doing, and what systems need to be put in place to support delivery? While the technical reports [15] (and this article) focus primarily on the first question, the main report [9] also discussed the second and third questions, and these are further discussed towards the end of this article.

We prioritize 28 effective CPS in British Columbia, Canada using an adapted version of the approach developed by HealthPartners Institute [12]. The policy goal is to guide decision-making by the BC Ministry of Health in initiating or expanding CPS within the province.

## Methods

### Definitions

A CPS is defined as any maneuver(s) pertaining to primary and early secondary prevention (i.e., immunization, screening, counselling and preventive medication/device) offered to the general (asymptomatic) population based on age, sex and risk factors for disease and delivered on a one-provider-to-one-client basis, with two qualifications: (i) the provider could work as a member of a care team or as part of a system tasked with providing, for instance, a screening service; and (ii) the client could belong to a small group (e.g. a family, a group of smokers) that is jointly benefiting from the service.

A clinically preventable burden (CPB) is defined as the total quality adjusted life years (QALYs) that could be gained if the CPS were delivered at recommended intervals to a BC birth cohort of 40,000 individuals (the approximate number of annual births in BC) over the years of life that the service is recommended. Cost-effectiveness (CE) is defined as the net cost per QALY gained.

### Selection of clinical preventive services for review

In 2006, the HealthPartners Institute published a study which ranked 25 evidence-based CPS on a scale of 2 (low priority) to 10 (high priority) [14]. Of the 25 CPS, 15 received a rank of 6 or higher. In 2008, we requested and received Excel-based models for 10 of the 15 CPS. The 10 models were adjusted to incorporate available BC-specific data in calculating CPB and CE. In the adjusted models, we also used the difference between no service and the best utilization rate for that CPS observed in high-income countries (see Table 1), rather than the 90% utilization rate assumed in the HealthPartners modelling [11]. This approach was chosen to better reflect actual benefits and costs associated with potentially achievable utilization rates.

**Table 1:** Potential clinical preventive services in BC. Summary of the applicable cohort, service frequency and coverage

***Clinical Preventive Service***	***Cohort / Timing***	***Frequency / Intensity***	***Estimated Coverage***		***Reference*** for BiW
			BC	BiW	
Screening / treatment for depression	Ages 12-18	Annually	Unknown	7.4%	[16]
Interventions to support breastfeeding	During pregnancy and after birth	Multiple sessions	Unknown	46%	[17]
Screening for obesity and referral to comprehensive, intensive behavioral intervention to promote improvement in weight status	Ages 6-17	Screening – at all appropriate primary care visits	Unknown	13%	[18]
		Intervention – attendance at > 70% of 10 2-h sessions	7.2%	7.2%	[19]
Preventing tobacco use	Ages 6-17	Annually	Unknown	53%	[20]
Application of fluoride varnish	On primary tooth at time of eruption (ages 1-5)	Every 6 months	Unknown	62%	[21]
Application of dental sealants	On permanent teeth at time of tooth eruption (ages 6-12)	4 times (on 1st and 2nd bicuspids & molars)	Unknown	59%	[22]
Screening / treatment for breast cancer	Ages 50-74	Every 2-3 years	52%	88%	[23]
Screening / treatment for cervical cancer (cytology-based)	Ages 25-69	Every 3 years	69%	88%	[24]
*Addition* of HPV-based cervical cancer screening	Ages 30-65	Every 5 years	0%	88%	[24]
Screening / treatment for colorectal cancer	Ages 50-74	FOBT every 2 years or sigmoidoscopy every 10 years	50%	76%	[25]
Screening / treatment for lung cancer	Ages 55 - 74 with a 30 pack-year smoking history	Annually for 3 consecutive years	Unknown	6%/60%	[26]
Screening / treatment for hypertension	Ages 18 and older	At least once every 2 years	Unknown	79%	[27]
Screening / treatment for CVD	Ages 40-74	Screening - once every 5 years	Unknown	48%	[28]
		Treatment - ongoing	Unknown	30%	[29, 30]
Screening / treatment for type 2 diabetes	Ages 18 and older – risk assessment	Every 3-5 years	Unknown	58%	[31]
	High risk for T2DM – blood glucose	Every 3-5 years	Unknown	80%	[﻿^32^]
	Very high risk for T2DM – blood glucose	Every year	Unknown	80%	
Screening / treatment for depression	Non-pregnant adults ages 18 and older	At least once	Unknown	12%	[33]
Screening / treatment for depression	Pregnant and postpartum females	At least once per birth by 8 weeks postnatally	Unknown	39%	[34]
Screening / treatment for osteoporosis	Females age 65	One-time	Unknown	58%	[35]
Screening / treatment for abdominal aortic aneurysm	Males age 65 who have ever smoked	One-time	Unknown	86%	[36]
Screening / treatment for HIV	Ages 15 – 65	Low risk – once	20%	45%	[37]
		Increased risk – every 3-5 years	20%	63%	[38]
		Very high risk – every year	20%	83%	[39]
		During all pregnancies	96%	97%	
Screening / treatment for chlamydia and gonorrhea	Sexually active females 24 years of age or younger	When sexual history reveals new or persistent risk factors since the last negative test	Unknown	55%	[40]
Screening / treatment for HCV	Adults born between 1945 & 1965	One-time	33%	48%	[41]
Screening and BCI for the prevention of sexually transmitted infections	All sexually active adolescents and adults who are at increased risk for STIs	30 min to ≥2 h of intensive behavioural counseling	Unknown	29%	[42]
Screening and BCI to prevent tobacco use	Ages 18 and older	Up to 90 min of total contact time, during multiple contacts	19%	51%	[43]
Screening and BCI to prevent alcohol misuse	Ages 18 and older	Screening – annually during primary care visit	Unknown	93%	[44]
		Screening – during pregnancy	Unknown	97%	[45]
		Brief intervention – three 10 min sessions	Unknown	41%	[46]
Screening for and management of obesity	Ages 18 and older	Screening – ongoing	Unknown	73%	[47]
		Management – at least one-time of 12-26 sessions in a year	Unknown	33%	[48]
Screening / treatment to prevent falls in the elderly	Community-dwelling elderly ages 65+	Screening for risk - every year	Unknown	18%	[49]
		Exercise - at least 150 min of moderate intensity / week	Unknown	Unknown	
		Vitamin D suppl. - 800 IU / day for at least 12 months	Unknown	61%	[50]
Routine aspirin use for the prevention of CVD disease and CRC	Age 50-69 with a 10% or greater 10-year CVD risk & at low risk of bleeding	Screening for CVD risk - at age 50-59	Unknown	33%	[51]
		Screening for bleeding risk - at age 50-59	Unknown	33%	
		Management - low-dose daily aspirin use for 10 years	Unknown	24%	[52]
Folic acid supplementation for the prevention of neural tube defects	Reproductive-age females	0.4 to 0.8 mg (400 − 800 μg) of folic acid daily	Unknown	34%	[50]

*Abbreviations*: *BC* British Columbia, *BiW* ‘Best in the World’, *BCI* Behavioural counselling intervention, *CVD* Cardiovascular disease, *HIV* Human immunodeficiency virus, *HCV* Hepatitis C virus, *CRC* Colorectal cancer

In 2013 the Expert Advisory Committee requested modelling for an additional 9 CPS, followed by 4 in 2015. Each subsequent year the Committee chose 2-4 CPS to (re)model, based on updated CTFPHC or USPSTF results. In 2018, the Committee requested a revision of the CPS modelled to date to incorporate more recent data. In this 2018 update, all costs were adjusted to 2017 Canadian dollars. For consistency, all models completed or revised since 2018 have continued to provide the cost / QALY in 2017 Canadian dollars. The Committee only considered inclusion of preventive maneuvers with a ‘strong’ or ‘conditional (weak)’ recommendation’ by the CTFPHC [53] or an ‘A’ or ‘B’ rating by the USPSTF [54].

In order to prevent duplicate evidence reviews, the Committee agreed to refer any recommendations regarding immunizations to the British Columbia Communicable Disease Policy Committee [55] and any recommendations regarding prenatal care, intrapartum care and immediate postpartum and postnatal care (up to 8 weeks) to Perinatal Services BC [56], thus these CPS are not considered in this manuscript.

Table 1 provides a summary of the 28 CPS reviewed in BC to date. Included in the table are the relevant cohort and the frequency with which the service is to be provided. In addition, an estimated rate of coverage for the service in BC and the best in the world (BiW) are provided.

The primary variables in each model include the effectiveness of the intervention, the quality of life (QoL) values associated with the relevant health state(s) and the costs associated with implementing the intervention and/or avoiding the relevant health state(s).

### Effectiveness of the intervention

Table 2 provides a summary of the effectiveness values (and the 95% CI) used in the modelling for each CPS. The effectiveness values are primarily based on evidence reviews completed for the CTFPHC or the USPSTF.

**Table 2:** Effectiveness values for each CPS used in modelling

***Clinical Preventive Service***	***Effectiveness (Range)***	***Reference***
Screening / treatment for depression (ages 12-18)	Cognitive behavioural therapy is associated with a clinically significant improvement in 12.1% of youth with MDD while fluoxetine is associated with a 25.7% (16.2 - 35.2%) improvement.	[57]
Interventions to support breastfeeding	Breastfeeding promotion interventions are associated with a 44% (13 - 84%) increase in long-term (≥6 months) exclusive breastfeeding.	[58]
Screening for obesity and referral to comprehensive, intensive behavioral intervention to promote improvement in weight status (children & youth)	Completion of a comprehensive intervention is associated with an 18.8% (6.1 – 40.2%) reduction in obesity.	[59, 60]
Preventing tobacco use (school-aged children & youth)	Interventions aimed at reducing smoking initiation / smoking cessation among non-smoking / smoking children and youth have an effectiveness of 18% (6 - 28%) and 34% (5 - 69%).	[61]
Application of fluoride varnish	The application of fluoride varnish reduces decayed, missing and filled teeth by 37% (24 - 51%).	[62]
Application of dental sealants	The application of dental sealants reduces decayed, missing and filled teeth by 84% at year 1, decreasing to 55% at year 9.	[63]
Screening / treatment for breast cancer	Screening mammography in women ages 50-74 leads to a reduction in breast cancer mortality of 21% (10 - 32%).	[64]
Screening / treatment for cervical cancer (cytology-based)	Cervical cancer screening in women ages 25-69 leads to a reduction in cervical cancer mortality of 35% (10 - 53%).	[65]
Addition of HPV-based cervical cancer screening	HPV-based screening is associated with a 55% (19 - 75%) reduction in the incidence of cervical cancers in females ages 30 – 64, when compared to cytology-based screening.	[66]
Screening / treatment for colorectal cancer (CRC)	Screening with gFOBT is associated with a reduction in mortality from CRC by 18% (8 - 27%) and the incidence of late stage CRC by 8% (1 - 15%). Screening with flexible sigmoidoscopy is associated a reduction in mortality from CRC by 26% (18 - 33%) and the incidence of late stage CRC by 27% (18 - 34%).	[67]
Screening / treatment for lung cancer	Screening for lung cancer is associated with a 19.6% (7.7 - 30.0%) reduction in mortality from lung cancer.	[68]
Screening / treatment for hypertension	Lowering blood pressure by 10/5 mmHg results in a 22% (17 -27%) reduction in cardiovascular events and a 41% (33 - 48%) reduction in cerebrovascular events.	[69]
Screening / treatment for cardiovascular disease	Statin therapy is associated with a 14% (7 - 20%) decreased risk of all-cause mortality, a 31% (12 - 46%) decreased risk of cardiovascular mortality, a 36% (29 - 43%) decreased risk of myocardial infarction and a 29% (18 - 38%) decreased risk of stroke.	[70]
Screening / treatment for type 2 diabetes	Screening / treatment for type 2 diabetes is associated with 5.2 (2.7 - 7.5) myocardial infarction events prevented, 8.0 (6.2 - 9.5) microvascular events prevented and 3.2 (1.0 - 5.8) premature deaths prevented per 1000 people screened.	[71]
Screening / treatment for depression (adults)	The use of ADM for major depression is associated with a 64% (12 - 85%) reduced risk of recurrent depression.	[72]
Screening / treatment for depression (pregnant and postpartum females)	Participation in programs involving depression screening leads to a 32% (18 - 59%) reduced risk of depression 3-5 months later.	[73]
Screening / treatment for osteoporosis	Long-term treatment compliance with bisphosphonates is associated with a 23% reduction in hip fractures and a 26% reduction in vertebral fractures.	[74, 75]
Screening / treatment for abdominal aortic aneurysm (AAA)	Screening and treatment for AAA is associated with a 115% (89 - 144%) increase in elective surgeries, a 48% (34 - 60%) reduction in emergency surgeries and a 42% (12 - 61%) reduction in AAA-related mortality.	[76]
Screening / treatment for HIV	The early initiation of antiretroviral therapy is associated with a 64% (25 - 96%) reduction in the transmission rate per person-year.	[77, 78]
Screening / treatment for chlamydia and gonorrhea	Screening reduces the lifetime risk of chronic pelvic pain, infertility and ectopic pregnancy by 41%.	[79]
Screening / treatment for HCV	The effectiveness of direct acting antiviral treatment in producing a sustained viral response (i.e., a cure) is 97% (95 - 99%).	[80–85]
Screening and BCI for the prevention of sexually transmitted infections	High intensity behavioural counselling interventions are associated with a 62% (40 - 76%) reduction in STI incidence in adolescents and a 30% (13 - 44%) reduction in STI incidence in adults.	[86]
Screening and BCI to prevent tobacco use (adults)	Quit rates improve from 10.9 to 28.0% (23.0 - 33.6%).	[87, 88]
Screening and BCI to prevent alcohol misuse (adults)	13.9% (8.7 - 16.1%) improvement in the proportion of adults achieving recommended drinking limits.	[89]
Screening for and management of obesity (adults)	20% (14 to 25%) of participants lost at least 5% of their body weight.	[90]
Screening / treatment to prevent falls in the elderly	Interventions involving exercise or physical therapy reduce falls in community-dwelling elderly by 13% (6 - 19%).	[91]
Routine aspirin use for the prevention of cardiovascular disease (CVD) and colorectal cancer (CRC)	Initiating low dose aspirin use for the primary prevention of CVD and CRC in adults aged 50 to 59 years who have a 10% or greater 10-year CVD risk, are not at increased risk for bleeding, have a life expectancy of at least 10 years, and are willing to take low-dose aspirin daily for at least 10 years reduces the risk of nonfatal myocardial infarction by 17% (6 – 26%), the risk of nonfatal stroke by 14% (2 – 24%), the incidence of colorectal cancer by 40% (24 – 53%) and the risk of death from CRC about 20 years later by 33% (14 – 48%).	[92, 93]
Folic acid supplementation for the prevention of neural tube defects (NTDs)	Daily supplementation during pregnancy results in a 69% (42 - 83%) reduction in NTDs.	[94]

*Abbreviations*: *BCI* Behavioural counselling intervention, *MDD* Major depressive disorder, *HPV* Human papillomavirus, *gFOBT* Guaiac fecal occult blood test, *ADM* Antidepressant medication

### Quality of life values used in the modelling

The primary source for QoL values were the disability weights developed for the Global Burden of Disease study [95, 96] adjusted to reflect the mean QoL of the age- and sex-specific population under consideration [97, 98]. If disability weights were not available in the Global Burden of Disease study, then meta-analysis or larger studies assessing the QoL for a specific health-related outcome were used.

The CPB was calculated based on benefits minus known harms. For example, we included harms associated with unnecessary follow-up interventions associated with false positive screening results. Harms also include a modest reduction in QoL associated with taking any medication for preventive purposes [99–101].

Table 3 provides an overview of the QoL values used in the modelling.

**Table 3:** Quality of life values used in the modelling

Health State (Definition or Duration)	QoL Reduction (Range)	Reference
Taking medication for prevention	0.0024 (0.00 – 0.0033)	[99–101]
Alcohol Use		
Binge drinking	0.123 (0.082 - 0.177)	GBD [96, 102, 103]
Hazardous alcohol use (3 to 4.5 drinks per day for males and 1.5 to 3 drinks per day for females)	0.179 (0.121 - 0.252)	
Harmful alcohol use (> 4.5 drinks per day for males and > 3 drinks per day for females)	0.304 (0.204 - 0.418)	
Atopic dermatitis / eczema	0.043 (0.026 – 0.065)	GBD
Cancer – Breast		
False-positive mammography result (4.7 days)	0.013	[104]
Diagnosis and treatment phase (3 months)	0.288 (0.193 - 0.399)	GBD, [102]
Metastatic phase (17.7 months)	0.451 (0.307 - 0.600)	
Remission	0.049 (0.031 - 0.072)	
Cancer – Cervical		
False-positive Pap smear (10 months)	0.046	GBD
Diagnosis and treatment for CIN (20 months)	0.066	
Diagnosis and treatment phase for cancer (4.8 months)	0.288 (0.193 - 0.399)	GBD, [105]
Metastatic phase (9.2 months)	0.451 (0.307 - 0.600)	
Remission	0.049 (0.031 - 0.072)	
Cancer – Colorectal		
Diagnosis and treatment phase (4 months)	0.288 (0.193 - 0.399)	GBD
Metastatic phase (9.7 months)	0.451 (0.307 - 0.600)	
Remission	0.049 (0.031 - 0.072)	
Cancer – Liver		
Diagnosis and treatment phase (4 months)	0.288 (0.193 - 0.399)	GBD
Metastatic phase (2.5 months)	0.451 (0.307 - 0.600)	
Remission	0.049 (0.031 - 0.072)	
Cancer – Lung		
Diagnosis and treatment phase (3.3 months)	0.288 (0.193 - 0.399)	GBD
Metastatic phase (4.5 months)	0.451 (0.307 - 0.600)	
Remission	0.049 (0.031 - 0.072)	
Cancer – Ovarian		
Diagnosis and treatment phase (3.2 months)	0.288 (0.193 - 0.399)	GBD
Metastatic phase (25.6 months)	0.451 (0.307 - 0.600)	
Remission	0.049 (0.031 - 0.072)	
Cardiovascular Disease - myocardial infarction (1 month)	0.100 (0.065 – 0.137)	GBD
Cardiovascular Disease - stroke	0.200 (0.134 - 0.265)	GBD, [106]
Childhood asthma	0.040 (0.024 – 0.060)	GBD, [107]
Chronic pelvic pain (5 years)	0.114 (0.078 - 0.159)	GBD, [79]
Dental caries		
Symptomatic dental caries	0.010 (0.005 - 0.019)	GBD
Severe tooth loss	0.067 (0.045 - 0.095)	
Depression		
Mild	0.145 (0.099 - 0.209)	[108]
Moderate	0.396 (0.267 - 0.531)	
Severe	0.658 (0.477 - 0.807)	
Diabetes – Type 2		
Uncomplicated	0.049 (0.031 - 0.072)	GBD
Diabetic neuropathy	0.133 (0.089 - 0.187)	
Ectopic pregnancy (4 weeks)	0.114 (0.078 - 0.159)	GBD, [79]
End-stage renal disease		
Chronic kidney disease	0.104 (0.070 - 0.147)	GBD
On dialysis	0.571 (0.398 - 0.725)	
Fetal alcohol spectrum disorder	0.50 (0.44 - 0.57)	[109]
Fetal alcohol syndrome	0.56 (0.48 - 0.63)	
Gastrointestinal bleeding (28 days)	0.265	[110]
Hepatitis C infection		
Non-cirrhosis (fibrosis stage 0-3)	0.088 (0.038 – 0.138)	[111, 112]
Compensated cirrhosis (fibrosis stage 4)	0.138 (0.088 – 0.188)	
Decompensated cirrhosis	0.188	
Liver transplant (1st year)	0.438	
Liver transplant (subsequent years)	0.163	
On-treatment	0.113 (0.063 – 0.163)	
HIV/AIDS		
Symptomatic HIV without anemia	0.274 (0.184 - 0.377)	GBD
Symptomatic HIV with mild anemia	0.277 (0.189 - 0.379)	
Symptomatic HIV with moderate anemia	0.312 (0.217 - 0.418)	
Symptomatic HIV with severe anemia	0.381 (0.269 - 0.505)	
AIDS with antiretroviral treatment (ART) without anemia	0.078 (0.052 - 0.111)	
AIDS with ART with mild anemia	0.081 (0.054 - 0.116)	
AIDS with ART with moderate anemia	0.125 (0.085 - 0.176)	
AIDS with ART with severe anemia	0.215 (0.148 - 0.295)	
Infertility		
Primary infertility	0.008 (0.003 - 0.015)	GBD
Secondary infertility	0.005 (0.002 - 0.011)	
Intellectual disability		
Borderline	0.011 (0.005 - 0.020)	GBD
Mild	0.043 (0.026 - 0.064)	
Moderate	0.100 (0.066 - 0.142)	
Profound	0.200 (0.133 - 0.283)	
Obesity		
Children / youth	0.026 (0.017 – 0.036)	[113, 114]
Adults	0.037 (0.024 – 0.049)	[115]
Osteoporosis		
Hip fracture (6 months)	0.355	[116]
Vertebral fracture (12 months)	0.050	[117]
Chlamydial or gonococcal infection – mild	0.006 (0.002 - 0.012)	GBD
Spina bifida		
Sacral lesion	0.34 (0.06 – 0.62)	[118]
Lower lumbar lesion	0.42 (0.22 – 0.62)	
Upper lumbar lesion	0.52 (0.25 – 0.78)	
Tobacco smoking		
Light (< 10 cigarettes / day)	0.031 (0.018 - 0.045)	[115]
Moderate (10-19 cigarettes / day)	0.033 (0.019 - 0.047)	
Heavy (≥20 cigarettes / day)	0.062 (0.042 - 0.082)	
Vision deficits		
Mild	0.003 (0.001 - 0.007)	GBD
Moderate	0.031 (0.019 - 0.049)	
Severe	0.184 (0.125 - 0.258)	
Blindness	0.187 (0.124 - 0.260)	

*Abbreviations*: *GBD* Global Burden of Disease

### Resource unit costs used in the modelling

In calculating CE, we included medical costs and costs to the individual. Medical costs included those associated with screening, counselling, pharmaceutical treatment and any follow-up diagnostic tests and treatments for both true- and false-positive findings. In the model assessing behavioural counselling and interventions for the prevention of alcohol misuse, we also included the costs associated with law enforcement, fire damage and motor vehicle collisions [119]. In the model assessing folic acid supplementation for all women of reproductive age, we also included the special education and developmental service costs associated with caring for a child with a neural tube defect [120]. While the definition of clinical prevention is independent of delivery mechanism or provider type, for costing purposes we chose to use a primary care physician’s office as the delivery mechanism when an established delivery mechanism was not in place in BC. We assumed that 50% of a 10-min visit would be required per CPS unless evidence indicated otherwise.

Costs to the individual include the value of a patient’s time required to travel to an appointment and receive both the CPS and needed follow-up procedures and is based on the average hourly wage rate in BC in 2017 plus 18% benefits [121]. If the ‘50% of a 10-minute visit’ assumption applied, then only 50% of a patient’s time costs were included in the modelling. Overall costs were reduced by potential savings resulting from avoided treatments or less intensive treatments associated with earlier-stage medical care.

When integrating unit cost information into the analyses, priority was given to information available from BC, followed by the rest of Canada, then other high income countries with health care systems similar to Canada (e.g. the UK and Australia) and finally to unit cost information from the US. All unit costs were converted to 2017 Canadian dollars using the Campbell and Cochrane Economics Methods Group and the Evidence for Policy and Practice Information and Coordinating Centre Cost Converter [122, 123]. If US health care unit costs were used, these costs were reduced by 29% to reflect the substantially higher unit costs (or prices) in the US compared to those in Canada for the same output [124–126].

Table 4 provides an overview of the unit costs used in the modelling.

**Table 4:** Unit costs used in the modelling

*In 2017 Canadian Dollars*		
Health State / Resource Unit	Unit Cost	Reference
Patient time costs	$29.69 / hour	[121]
Office visit to a General Practitioner	$34.85	[127]
GP follow-up phone call or email correspondence	$15.00	
Abdominal aortic aneurysm		
Emergency repair surgery	$46,853	[128–132]
Elective open surgery	$45,998	
Elective endovascular aneurysm repair surgery	$36,039	
Alcohol Use		
Low	$91(F) $195(M) / year	[133, 134]
Hazardous (3 to 4.5 drinks per day for males and 1.5 to 3 drinks per day for females)	$708(F) $1238(M) / year	
Harmful (> 4.5 drinks per day for males and > 3 drinks per day for females)	$2925(F) $3133(M) / year	
Fetal alcohol spectrum disorder lifetime cost	$1,118,811	[135]
Fetal alcohol syndrome lifetime cost	$1,664,074	
Atopic dermatitis / eczema lifetime costs	$3420	[136]
Cancer – breast		
Mammogram	$79	[137]
Biopsy	$386	[104]
Radiotherapy	$5233	[138]
Breast conserving surgery	$5152	
Mastectomy	$7260	
Acute care phase of fatal cancer	$47,230	[139, 140]
First year costs for survivors	$22,695	
Ongoing annual costs for survivors	$1753	[141]
Cancer – cervical		
Conventional cytology screen	$70	[142–144]
HPV test	$96	[145]
Colposcopy with biopsy	$251	[142, 143]
Treatment for a precancerous lesion	$1216	[142, 143]
Acute care phase of fatal cancer	$46,603	[139]
First year costs for survivors	$20,258	
Ongoing annual costs for survivors	$821	[146]
Cancer – colorectal		
FIT test	$14.74	BC Medical Services Plan (MSP)
Colonoscopy	$667	BC MSP
Acute care phase of fatal cancer	$49,197	[139]
First year costs for survivors	$40,080	
Ongoing annual costs for survivors	$3687	[147]
Cancer – liver		
Acute care phase of fatal cancer	$30,922	[139]
First year costs for survivors	$36,708	
Ongoing annual costs for survivors	$6287	[147]
Cancer – lung		
LDCT screening exam	$198	[148]
Follow-up chest radiograph	$67	
Follow-up chest CT	$164	
Follow-up PET/CT scan	$1399	
Percutaneous biopsy – CT-guided	$1083	
US-guided	$682	
Bronchoscopy without biopsy	$747	
Bronchoscopy with biopsy	$804	
Mediastinoscopy	$976	
Thoracoscopy	$16,814	
Thoracotomy	$18,689	
Acute care phase of fatal cancer	$37,046	[139]
First year costs for survivors	$33,523	
Ongoing annual costs for survivors	$7575	[147]
Cancer – ovarian		
Acute care phase of fatal cancer	$51,914	[139]
First year costs for survivors	$33,256	
Ongoing annual costs for survivors	$7889	[147]
Cardiovascular disease		
Full lipid profile	$21.31	[149]
Annual cost of statin medication	$135	[150]
Cardiovascular Disease - myocardial infarction		
Acute care phase of a fatal MI	$15,536	[151]
First year costs for survivors	$33,934	[152]
Ongoing annual costs for survivors	$2278	
Cardiovascular Disease – stroke		
First year costs for survivor	$21,139	[152, 153]
Ongoing annual costs for survivors	$6246	
Childhood asthma		
Lifetime per case	$5230	[154]
Childhood leukemia		
Lifetime per case	$134,920	[155]
Dental caries		
Topical fluoride application	$10.61	[156]
Pit and fissure sealant application (1st/subsequent per quadrant)	$19.74 / $10.83	
Amalgam restoration	$93	
Day surgery for dental cavities	$1884	[157]
Depression		
Antidepressant medication (ADM) / year (adults)	$438	[158]
ADM / year (adolescents)	$368	[159]
Group therapy (CBT session for 12 adolescents)	$241	[160]
Group therapy (CBT session for 8 pregnant females)	$269	
Annual health care costs attributable to depression (adolescents)	$5251	
Suicide attempt	$9056	[161–163]
Completed suicide	$8233	
Diabetes – Type 1 lifetime cost	$76,598	[155]
Diabetes – Type 2		
Cost per A1C test	$6.09	[164]
Blindness (annual cost)	$2330	[165]
Lower extremity amputation (surgery/annual cost)	$33,642 / $1396	[166]
End-stage renal disease annual cost	$86,278	
Gastrointestinal bleeding (per hospitalization)	$6425	[167]
Gastrointestinal / lower respiratory tract infection (per infection)	$462	[168]
Hepatitis C infection		
Incremental annual health care cost		
HCV infection (non-cirrhosis stages f0 to f3)	$400	[169, 170]
Compensated cirrhosis (stage f4)	$843	
Decompensated cirrhosis	$15,284	
Cost of direct-acting antivirals	$13,500	[171–173]
Complete blood count	$10.96	BC MSP
Thyroid stimulating hormone	$9.90	BC MSP
Renal panel	$31.52	BC MSP
Liver transplant (first year / annual)	$162,901 / $9654	[174]
HIV / AIDS		
Annual cost of ART	$9490	[158]
Annual direct medical costs (excluding medications)		
Asymptomatic HIV	$1889	[175]
Symptomatic HIV	$2843	
AIDS	$10,900	
Hypertension		
Annual cost of antihypertensive medication	$193	[158]
Intellectual disability lifetime costs	$270,345	[176]
Lower extremity amputation (surgery / ongoing annual)	$33,642 / $1396	[166]
Neural tube defects		
Folic acid supplementation (annual costs)	$15.70	[177]
Spina bifida (lifetime costs)	$801,991	[120]
Anencephaly live birth	$4399	[178, 179]
Obesity		
Excess annual medical care costs	$698 (M) / $953 (F)	[133]
Structured behavioural intervention (per child/youth)	$7681	[180]
Structured behavioral intervention (per adult)	$607	[181–183]
Osteoporosis		
Bone density scan	$111	[184]
Annual cost of medication	$188	[159]
Hip fracture annual costs	$62,152	[185]
Vertebral fracture annual costs	$25,965	
Otitis media per case	$251	[186]
Sexually transmitted infections		
Group behavioural counselling intervention (session for 5 individuals)	$487	Calculated
Direct medical costs per infection		
Chlamydia	$229	[187]
Gonorrhea	$169	
Hepatitis B virus	$2536	
HIV	$289,543	
Human papilloma virus	$112	
Herpes simplex virus type 2	$632	
Syphilis	$674	
Tobacco smoking		
Light (< 10 cigarettes / day)	$785 / year	[133]
Moderate (10-19 cigarettes / day)	$1386 / year	
Heavy (≥20 cigarettes / day)	$2050 / year	
Smoking cessation aids per quit attempt	$272	[188]

### Sensitivity analysis

One-way sensitivity analysis, in which each major variable or assumption in the model was modified, was performed to assess the robustness of the results. We used 95% confidence intervals (CIs) to inform the range for these variables in our sensitivity analyses when the 95% CIs were available. QALYs are not discounted in calculating CPB but both QALYs and costs are discounted by 1.5% in calculating CE, with this rate varied from 0 to 3% in the structural sensitivity analysis [189, 190].

Table 5 presents the range of CE estimates for each CPS together with key variables and the values for the key variables used in the base model and the sensitivity analyses.

**Table 5:** Potential clinical preventive services in BC. Range of cost-effectiveness estimates based on one-way sensitivity analysis

Clinical Preventive Services	CE^**a**^ (1.5% Discount Rate)			Key Variable(s)	Base Value	Range	
	Base	Range					
Screening for Asymptomatic Disease or Risk Factors - Youth							
Screening for depression	$28,215	$21,555	$45,994	Reduction in quality of life due to depression	31%	15%	45%
Behavioural Counseling Interventions - Children/Youth							
Interventions to support breastfeeding	($9021)	($14,757)	$19,699	% of women attending interventions who exclusively breastfeed at 6 months	44%	13%	84%
Growth monitoring and healthy weight management in children and youth	$29,436	$10,148	$524,527	Length of time that avoided costs accrue	Lifetime	10 Years	
Preventing tobacco use (school-aged children & youth)	($7349)	($10,083)	$23,905	% of smokers who cease as a result of intervention(s)	34%	5%	69%
Preventive Medication / Devices - Children							
Fluoride varnish	$43,038	$16,391	$86,076	Change in quality of life due to improved oral health	0.01	0.005	
				Frequency of fluoride varnish application	Every 6 months		Annually
Dental sealants	($24,690)	($32,248)	($17,132)	Cost of dental fillings	$92.75	$83.10	$102.40
Screening for Asymptomatic Disease or Risk Factors - Adults							
Screening for breast cancer	$19,720	$11,659	$45,514	% reduction in breast cancer deaths as a result of mammogram screening	21%	10%	32%
Screening (cytology-based) for cervical cancer	$25,542	$13,818	$99,328	Effectiveness of screening in reducing cervical cancer deaths / incidence	35% / 44%	10% / 25%	53% / 58%
*Addition* of HPV-based cervical cancer screening	($21,556)	($16,414)	($23,377)	% improvement in HPV-screening effectiveness vs. cytology-based screening	55%	19%	75%
Screening for colorectal cancer	$47,265	$32,923	$82,979	Effectiveness of screening in preventing colorectal cancer deaths	gFOBT - 18%	8%	27%
					Colonoscopy - 26%	18%	33%
Screening for lung cancer	$2240	$1228	$9206	% of lung cancer deaths avoided with screening	19.6%	7.7%	30.0%
Screening for hypertension	$15,254	$9314	$24,485	Effectiveness of drug treatment in reducing cardiovascular and cerebrovascular events	Cardio - 22%	17%	29%
					Cerebro - 41%	33%	48%
Screening for cardiovascular disease risk and treatment (with statins)	$3223	$1458	$7849	Effectiveness of drug treatment in reducing all-cause mortality, myocardial infarction and stroke	Mortality - 14%	7%	20%
					MI - 36%	29%	43%
					Stroke - 29%	18%	38%
Screening for type 2 diabetes mellitus (T2DM)	($3121)	($6348)	$1121	Proportion of office visit for screening	50%	33%	
Screening for depression in general adult population	Dominated						
Screening for depression in pregnant and postpartum women	$23,042	$11,149	$43,255	% reduction in depression risk due to screening	32%	18%	59%
Screening for osteoporosis	($29,412)	($43,257)	$38,997	Change in the effectiveness of screening / treatment			
				Hip fracture reduction rate	23%	8%	$36
				Vertebral fracture reduction rate	26%	12%	$38
Screening for abdominal aortic aneurysm (AAA)	$11,995	$9328	$38,251	Change in the relative risk of AAA-related mortality	0.58	0.39	0.88
Screening for Sexually Transmitted Infections and Blood Borne Pathogens - Adults							
Screening for human immunodeficiency virus	$16,434	($12,463)	$80,739	% reduction in HIV transmission rate with early antiretroviral therapy	64%	25%	96%
Screening for chlamydia and gonorrhea	$57,174	$37,189	$234,414	Effectiveness of screening in reducing pelvic pain, infertility and ectopic pregnancy	41%	10%	
Screening for hepatitis C virus	$3427	$2570	$5141	Probability of cirrhosis in Hepatitis C Virus positive individuals	15%	10%	20%
Behavioural Counseling Interventions - Adults							
Prevention of sexually transmitted infections (STIs)	$10,267	$6921	$22,513	Effectiveness of high intensity behavioural counselling interventions in reducing the incidence of STIs in adolescents and adults	Adolescents - 62%	40%	74%
					Adults - 30%	13%	44%
Counselling and interventions to prevent tobacco use	($1863)	($3441)	$779	Quit rate for smoking as a result of intervention	28.0%	23.0%	33.6%
Screening and behavioural counseling interventions to reduce unhealthy alcohol use	$9609	($375)	$23,676	Frequency of screening	Annual		Every 5 yrs
				Effectiveness of counselling in changing behaviour	13.9%	8.7%	
Screening for and management of obesity	$12,160	$5682	$28,565	Frequency of measuring of height / weight, physical activity and diet advice	Every 2 yrs	Annual	Every 3 yrs
Preventing falls	$35,213	$13,950	$77,738	Cost of exercise per hour	$5.00	$0.00	$15.00
Preventive Medication / Devices - Adults							
Routine aspirin use for the prevention of cardiovascular disease (CVD) and colorectal cancer	$2302	-$1189	$24,255	Effectivenes of aspirin in reducing risk of cardiovascular disease, cerebrovascular disease, and colorectal cancer incidence and death	Cardio - 17%	6%	26%
					Cerebro - 14%	2%	24%
					CRC Incidence - 40%	24%	53%
					CRC Mortality - 33%	14%	48%
Folic acid supplementation for the prevention of neural tube defects	$195,379	$88,410	$431,770	Frequency of advice on folic acid supplementation	Annual		Every 3 yrs

^a^*CE* Cost-effectiveness

## Results

Table 6 provides a summary of the CPB and CE associated with each of the 28 CPS maneuvers. The CPB columns identify the clinically preventable burden (in terms of QALYs) that is being achieved in BC based on current coverage, and the potential CPB if the best coverage rate in the world (BiW) is achieved. Note that coverage rates in BC are unknown for 21 of the 28 (75%) maneuvers. The CE columns identify the cost-effectiveness ratio associated with a service stated in terms of the cost per QALY, using both a 1.5% and a 0% discount rate. The top interventions in terms of CPB are screening for hypertension and screening for cardiovascular disease risk and treatment that would prevent 11,587 and 9370 QALYs lost per 40,000 individuals, respectively. The top interventions in terms of CE are screening women 65 and older for osteoporosis and the application of dental sealants on permanent teeth at the time of tooth eruption, which provide cost savings of $29,412 and $24,690 per QALY (with 1.5% discount), respectively.

**Table 6:** Potential clinical preventive services in BC. Summary of the clinically preventable burden and cost-effectiveness

Clinical Preventive Services	CPB^**b**^ (0% Discount)			CE^**c**^ (% Discount)	
	B.C.	‘BiW’^a^	Gap	1.5%	0%
Screening for Asymptomatic Disease or Risk Factors - Youth					
Screening for depression	Unknown	222		$28,215	$27,331
Behavioural Counseling Interventions - Children/Youth					
Interventions to support breastfeeding	Unknown	5002		($9021)	($11,966)
Growth monitoring and healthy weight management in children and youth	196	196	0	$29,436	$18,148
Preventing tobacco use (school-aged children & youth)	Unknown	4123		($7349)	($9538)
Preventive Medication / Devices - Children					
Fluoride varnish	Unknown	150		$43,038	$43,038
Dental sealants	Unknown	157		($24,690)	($29,320)
Screening for Asymptomatic Disease or Risk Factors – Adults					
Screening for breast cancer	703	1189	486	$19,720	$18,326
Screening (cytology-based) for cervical cancer	1153	1471	318	$25,542	$26,980
*Addition* of HPV-based cervical cancer screening	0	655	655	($21,556)	($19,264)
Screening for colorectal cancer	1141	1734	593	$47,265	$44,213
Screening for lung cancer	Unknown	1745		$2240	$2080
Screening for hypertension	Unknown	11,587		$15,254	$10,760
Screening for cardiovascular disease risk and treatment (with statins)	Unknown	9370		$3223	$1392
Screening for type 2 diabetes mellitus (T2DM)	Unknown	3494		($3121)	($3453)
Screening for depression in general adult population	Unknown	-8		Dominated	
Screening for depression in pregnant and postpartum women	Unknown	109		$23,042	$10,140
Screening for osteoporosis	Unknown	91		($29,412)	($34,145)
Screening for abdominal aortic aneurysm	Unknown	340		$11,995	$9973
Screening for Sexually Transmitted Infections and Blood Borne Pathogens - Adults					
Screening for human immunodeficiency virus	Unknown	360		$16,434	$16,434
Screening for chlamydia and gonorrhea	Unknown	143		$57,174	$53,410
Screening for hepatitis C virus	2695	3920	1225	$3427	$2810
Behavioural Counseling Interventions - Adults					
Prevention of sexually transmitted infections (STIs)	Unknown	3285		$10,267	$10,267
Counselling and interventions to prevent tobacco use	3730	5944	2214	($1863)	($3344)
Screening and behavioural counseling interventions to reduce unhealthy alcohol use	Unknown	5035		$9609	$9258
Screening for and management of obesity	Unknown	2287		$12,160	$11,140
Preventing falls	Unknown	429		$35,213	$35,213
Preventive Medication / Devices – Adults					
Routine aspirin use for the prevention of cardiovascular disease (CVD) and colorectal cancer	Unknown	1098		$2302	$411
Folic acid supplementation for the prevention of neural tube defects	Unknown	95		$195,379	$113,155

^a^*’BiW’* Best in world, ^b^*CPB* Clinically preventable burden, ^c^*CE* Cost-effectiveness

**Fig. 1:**
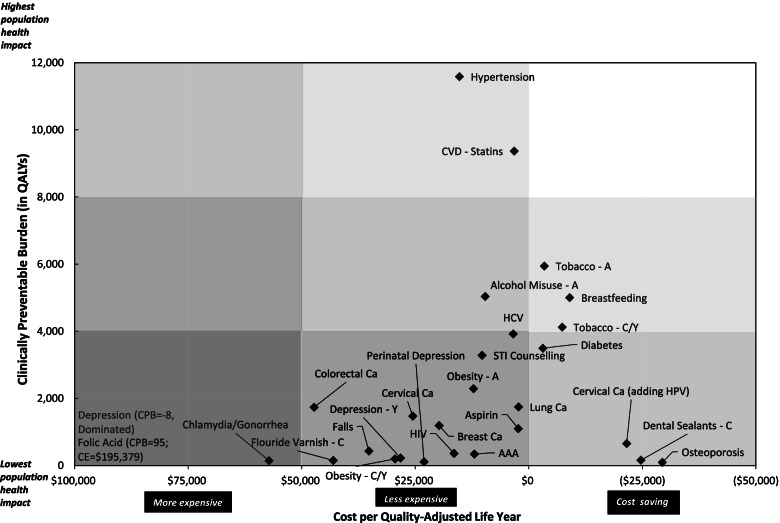
Establishing Priorities among Effective Clinical Preventive Services in BC. Combining Clinically Preventable Burden and Cost-Effectiveness Summary Results

The results for CPB and CE are displayed together in Fig. 1. The figure is divided into nine segments; from the lowest to highest population health impact and from more expensive to cost-saving. By arranging CPB and CE in this manner, services in the upper right segment have the most favourable combination of CPB and CE while services in the lower left segment have the least favourable combination. While no CPS fall into the high population impact / cost-saving segment, services that fall into the moderate population impact / cost-saving or high population impact / less expensive segments include prevention and cessation of tobacco use in both children/adolescents and adults; initiatives to improve exclusive breastfeeding to 6 months of age; screening for and treatment of hypertension; and screening for cardiovascular disease risk factors and the appropriate initiation of statins. Three additional CPS approach the moderate population impact / cost-saving or high population impact / less expensive segments, namely, alcohol misuse screening and brief counseling, one-time screening for HCV infection in BC adults born between 1945 and 1965, and screening for type 2 diabetes. Screening for osteoporosis, the application of dental sealants and the addition of screening for the human papillomavirus to cytology-based screening for cervical cancers, the CPS with the highest cost savings per QALY, fell in the lowest segment for population health impact.

**Fig. 2:**
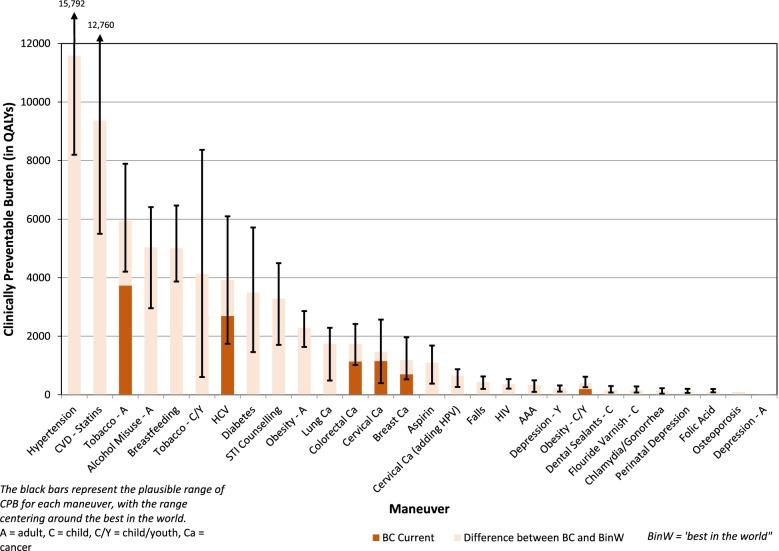
Clinically Preventable Burden Based on Providing Clinically Effective Services to a BC Birth Cohort of 40,000 0% Discount Rate

**Fig. 3:**
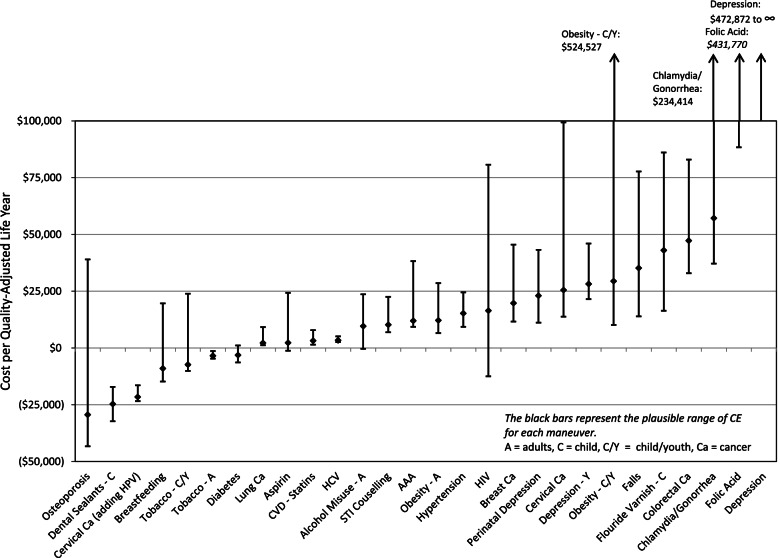
Cost Effectiveness Based on Providing Clinically Effective Services to a BC Birth Cohort of 40,000. Best Estimate and Plausible Range of Cost/QALY. 1.5% Discount Rate

The CBP and CE estimates were fairly stable for most CPS, but varied greatly for some (see Figs. 2 and 3). For example, for the CPS of primary care interventions aimed at smoking cessation among children and adolescents, the estimate of CBP varied from 606 to 8367 QALYs, and the cost-effectiveness estimates ranged from a **cost** of $23,905 / QALY to a **savings** of $10,083 / QALY.

Other CPS with large variation in the CE were screening women 65 and older for osteoporosis, screening adolescents and adults aged 15 to 65 years for infection with the human immunodeficiency virus, growth monitoring and healthy weight management in children and youth and screening females less than 30 years of age at increased risk for infection with chlamydia and gonorrhea. The most common reason for this variation is the uncertainty associated with the effectiveness of the intervention (see Table 5).

## Discussion

We have assessed the clinically preventable burden and cost-effectiveness ratio of 28 clinical preventive services in BC, Canada and found that the services with the highest population impact and best value for money include services that address tobacco use in adolescents and adults, exclusive breastfeeding, and screening for hypertension and other cardiovascular disease risk factors followed by appropriate pharmaceutical treatment. Three additional CPS approach these high-value CPS, namely alcohol misuse screening and brief counseling, one-time screening for hepatitis C virus infection in BC adults born between 1945 and 1965, and screening for type 2 diabetes.

Research by the HealthPartners Institute also established that the two CPS addressing tobacco use in the US were the highest priority preventive services [11]. Despite historically low rates of tobacco use in BC, which are the lowest of any province in Canada [191], tobacco use continues to exert an important influence on the ill-health of the population. Of greater concern is the varying range in the rate of tobacco use in the different geographic regions within BC, from 8.8 to 21.3% in 2011/12 [192]. This suggests the need for equity-focused CPS interventions based on the principle of proportionate universality; preventive services should be universally available, but concentrated on populations with higher rates of the condition or behaviour being addressed [193].

Our analysis also indicates the high value of interventions to support exclusive breastfeeding to 6 months. There are substantial health benefits for both the infant and mother associated with exclusive breastfeeding [58, 120].

Research by the HealthPartners Institute assigned a high value to addressing obesity-related behaviours. Our results for these CPS are more modest, likely due to assumptions about potential coverage rates. Based on the best available information on utilization rates from high-income countries, we assumed that only 7.2% of children [194, 195] and 33% of adults [47] with obesity would complete the multiple sessions over a 1 year period required to achieve an effective intervention [ 11, 49]. These coverage rates compare to the assumption of 90% included in the HealthPartners Institute analysis [196].

The limitations associated with this analysis are common to all modeling studies [197]. Models use data from a variety of sources and the results are only as good as the underlying data. By nature, models also simplify the causal chain so the assumptions made in doing so can have an important impact on results.

Another limitation is the ability to find BiW intervention rates for each CPS. Despite significant effort searching the academic and grey literature, together with expert input, it is not possible to determine whether or not the estimated BiW rates used in the models truly are the BiW. Furthermore, newer CPS such as lung cancer screening may currently have low screening rates that will improve over time. In this scenario, despite a BiW published screening rate of 6% [26], we assumed that the rate for lung cancer screening would eventually approximate rates associated with other cancer screening programs in BC (60%).

The definition of a CPS is independent of delivery mechanism(s) or provider type(s). Determining the most suitable delivery mechanism or provider type for each service is determined in subsequent phases of the policy cycle where decisions are made on whether and, if so, how to implement the CPS. In order to estimate the costs of providing the service and for consistency and comparability between the various CPS, we chose to use a general physician’s office as the delivery mechanism and provider type if an established delivery mechanism is not currently in place. Further work has started in determining if the effectiveness of the intervention changes based on who provides the intervention. For example, evidence indicates that brief behavioural counselling interventions to reduce unhealthy alcohol use are equally effective if provided by nurses, physicians or counsellors / mental health clinicians [198].

The results generated through this process provide a transparent and evidence informed approach to making decisions for the delivery of CPS. It is a key step in determining which CPS should be priorities for BC and is essential for creating a business plan for implementation. These results, however, should not be used in isolation. Actual changes to service provision should be undertaken only when this analysis, a detailed business plan and budget impact analysis are part of the process. These supplementary analyses are important in addressing further questions required in decision-making, such as the feasibility and total costs of enhancing current services or implementing new services and the potential impact on related services.

In BC, this work by the CPPR led to the province adopting a Lifetime Prevention Schedule (LPS), publishing a LPS Practice Guide and providing regular update reports [15]. The work by the CPPR was also a key building block in developing and implementing a preventive services incentive fee for family physicians in the province (the Personal Health Risk Assessment fee [199]), which we believe is unique in Canada. This analysis has also been instrumental in the decision to launch a lung cancer screening program in BC [200]. Finally, the development of business cases to enhance screening for tobacco smoking and alcohol misuse followed by a behavioural counselling intervention are currently in process.

## Conclusion

While the results noted above enable us to say with some confidence what is worth doing, the second and third questions asked in our original report remain important: What is the best way to deliver these services and by whom, and what supporting systems need to be put in place to ensure high and equitable coverage of cost-effective services with a moderate to high population health impact? While this discussion has started in BC and key decisions are being made, more remains to be done, both in BC and across Canada. After all, if a CPS is worth doing, it is worth doing well.

## Data Availability

A write-up of the detailed modelling approach including all assumptions and results for each individual model are available online at the British Columbia – Lifetime Prevention Schedule website (https://www2.gov.bc.ca/gov/content/health/about-bc-s-health-care-system/health-priorities/lifetime-prevention). In addition to the detailed results for each model included in the “LPS Update Report”, this website also includes a “Reference and Key Assumptions” document that details the methodology behind the Lifetime Prevention Schedule as well as key assumptions used throughout the process. The Excel-based detailed models are available from the lead author upon reasonable request and with permission of the British Columbia Ministry of Health.

## Abbreviations

BC: British Columbia
CPS: Clinical preventive services
CPB: Clinically preventable burden
CE: Cost-effectiveness
CTFPHC: Canadian Task Force on Preventive Health Care
USPSTF: United States Preventive Services Task Force
QALYs: Quality adjusted life years
CPPR: Clinical Prevention Policy Review
QoL: Quality of life
CIs: Confidence intervals
BiW: Best coverage rate in the world
